# A post-marketing observational study of ramucirumab in patients with gastric cancer in Japan

**DOI:** 10.1007/s10120-021-01199-0

**Published:** 2021-05-28

**Authors:** Yucherng Chen, Taeko Katayose, Soshi Nagaoka, Yongzhe Piao, Kensei Yamaguchi, Hiroya Asou

**Affiliations:** 1 Eli Lilly Japan K.K., Lilly Plaza One Building, 5-1-28 Isogamidori, Chuo-Ku, Kobe, Hyogo 651-0086 Japan; 2 Department of Gastroenterological Chemotherapy, Cancer Institute Hospital of Japanese Foundation for Cancer Research, Tokyo, Japan; 3 Saitama Cancer Center, Saitama, Japan

**Keywords:** Ramucirumab, Post-marketing surveillance, Gastric cancer

## Abstract

**Background:**

This study evaluated the safety and effectiveness of ramucirumab monotherapy and combination therapy for advanced gastric cancer in the real-world setting.

**Methods:**

This single-arm, prospective, multicenter, non-interventional, observational, post-marketing study was conducted in Japan from August 2015 to March 2019. Patients with unresectable advanced or recurrent gastric cancer and newly prescribed ramucirumab were followed for up to 12 months after first treatment. Data on adverse events and survival were collected via Electronic Data Capture.

**Results:**

Of 687 enrolled patients, 658 were eligible for analysis. Most patients received either ramucirumab monotherapy (123/658; 18.7%) or ramucirumab plus paclitaxel combination therapy (528/658; 80.2%). The majority of patients reported ≥ 1 adverse events in both the combination therapy (any grade, 479/528; 90.7%; ≥ Grade 3, 321/528; 60.8%) and monotherapy groups (any grade, 77/123; 62.6%; ≥ Grade 3, 42/123; 34.2%). The most common any grade adverse events were neutropenia (combination: 49.6%; monotherapy: 8.9%), fatigue (combination: 19.5%; monotherapy: 13.8%), and decreased appetite (combination: 18.2%; monotherapy: 10.6%). Grade 5 adverse events were reported in 4 patients, including metastases to meninges, pneumonia aspiration, death, and gastric perforation; of these, gastric perforation was deemed treatment-related. Median survival time was 5.7 months (95% confidence interval: 4.1–6.8 months) following monotherapy and 11.0 months (95% confidence interval: 9.8–12.2 months) following combination therapy.

**Conclusions:**

This analysis adds to the limited data available on ramucirumab use in a real-world setting, demonstrating similar safety and effectiveness for ramucirumab in treating advanced gastric cancer in routine clinical practice in Japan to that of global clinical trials.

**Supplementary Information:**

The online version contains supplementary material available at 10.1007/s10120-021-01199-0.

## Introduction

Gastric cancer is the fifth most commonly diagnosed and third most deadly cancer globally [1]. Incidence rates are higher in Asian countries, including Japan, where gastric cancer is the second most common cancer [2]. Combination therapies with platinum and fluoropyrimidine agents are currently the standard first-line treatment for unresectable advanced gastric cancer [3]; however, survival is poor and second-line treatment options are limited.

In global phase 3 studies of gastric cancer, ramucirumab, a human IgG1 monoclonal antibody against vascular endothelial growth factor receptor-2 [4], has demonstrated significant survival benefits and an acceptable safety profile in the second-line setting. In patients with advanced gastric cancer who had progressed on platinum/fluoropyrimidine-based therapies, progression-free survival and overall survival were significantly improved with ramucirumab, both as a monotherapy and in combination with paclitaxel (ramucirumab + paclitaxel) [5, 6]. Globally, ramucirumab + paclitaxel is currently a preferred second-line treatment option for patients with advanced gastric cancer who have progressed after first-line chemotherapy [3, 7, 8] and is the only recommended ‘evidence level A’ second-line regimen in the current Japanese gastric cancer treatment guidelines [9]. However, the majority of data on ramucirumab treatment is from clinical trials, and real-world data on ramucirumab treatment in gastric cancer patients are limited [10–13].

The objective of this study was to evaluate the safety and effectiveness of ramucirumab treatment in patients with unresectable advanced or recurrent gastric cancer in routine clinical practice in Japan. Data for ramucirumab monotherapy and ramucirumab combination therapy are described separately. Given the widespread use of ramucirumab for the treatment of advanced gastric cancer, we anticipate that these data will be referential and informative not only for clinicians in Japan but also for healthcare providers worldwide.

## Materials and methods

### Patients

This study includes patients with unresectable advanced/recurrent gastric cancer who were being treated for the first time with ramucirumab under routine clinical practice in Japan. This study had no exclusion criteria. Patients included in this analysis received ≥ 1 dose of ramucirumab. The study was conducted in compliance with the Good Post-marketing Study Practice in Japan.

### Study design and data collection

This was a single-arm, prospective, multicenter, non-interventional, observational, post-marketing study conducted from August 17, 2015 (first-patient visit) to March 18, 2019 (database lock). Data were reported by investigators at participating medical facilities via a case report form (CRF) in an Electronic Data Capture system. Patients were observed for up to 12 months following the first dose of ramucirumab. For safety data, follow-up observation was carried out for 30 days after discontinuation of ramucirumab or post-discontinuation treatment was started, whichever was earlier. For survival data, follow-up continued past 12 months, if allowed by investigators.

All treatment decisions were at the investigator’s discretion. The approved adult dosage for gastric cancer in Japan is 8 mg/kg (body weight) of ramucirumab, administered intravenously over 60 min every 2 weeks, with adjustments by the investigator allowed as deemed necessary for the patient’s condition.

Data were collected on patient demographic and clinical characteristics, treatment history, concomitant therapies, safety, and survival. Adverse events (AEs), serious adverse events (SAEs), and adverse events of special interest (AESIs) were classified using Medical Dictionary for Regulatory Activities, Version 21.1, preferred terms (consolidated terms where indicated) and graded based on the National Cancer Institute Common Terminology Criteria for Adverse Events, Version 4.0. SAEs were defined according to the International Council for Harmonisation of Technical Requirements for Pharmaceuticals for Human Use guideline E2A. Survival status was defined from the date of first ramucirumab treatment to the date of death (any cause) or until the last day of observation, as confirmed in the CRF.

### Statistical analysis

Continuous variables were summarized using mean (standard deviation [SD]) and median. Categorical variables were summarized using frequency and incidence. If baseline data were missing, the patient was counted as ‘not described’ or ‘not measured’ in the relevant categories. Data were analyzed for the overall study population and by treatment, categorized as ramucirumab monotherapy, ramucirumab + paclitaxel therapy, other ramucirumab combination therapy, and unspecified ramucirumab therapy (monotherapy/combination therapy details not provided by investigator). Patients were categorized in the ramucirumab + paclitaxel group if paclitaxel was administered on the same day as ramucirumab ≥ 1 time during the study period and in the other ramucirumab combination therapy group if another drug(s) (but no paclitaxel) was administered concomitantly with ramucirumab. Data for the other ramucirumab combination and unspecified ramucirumab therapy groups are included in the overall analysis population and are not separately summarized due to small sample size. Subgroup analyses examined treatment outcomes relative to selected baseline characteristic categories to determine factors affecting ramucirumab safety and effectiveness. Analyses were exploratory in nature. For comparisons between treatment groups, no covariate adjustments were made and *p* values are not reported. Survival status was estimated using the Kaplan–Meier method [14], including median survival time and associated 95% confidence intervals (CIs).

## Results

### Patients and treatments

In total, 687 patients were enrolled in the study. CRFs were collected for 683 patients (Online Resource 1). Of these, 25 patients were excluded, the majority of whom (*n* = 16; 64%) because they did not receive ramucirumab. A total of 658 patients were included in both the safety and the effectiveness analysis datasets, of which 123 (18.7%) received ramucirumab monotherapy, 532 (80.9%) received ramucirumab in combination with another anti-cancer drug, and 3 (0.5%) received unspecified ramucirumab treatment. Paclitaxel was the most common anti-cancer drug used concomitantly (528/532; 99.2%) whereas four patients (0.8%) received ramucirumab in combination with another anti-cancer drug (≥ 1 of irinotecan hydrochloride hydrate, fluorouracil, docetaxel, and/or cisplatin).

### Demographics and baseline characteristics

The majority of patients were male (*n* = 463; 70.4%) with a median age of 68.0 years (Table 1). Patients were diagnosed with metastatic (*n* = 652; 99.1%) cancer of the stomach (*n* = 609; 92.6%) or gastroesophageal junction (*n* = 48; 7.3%), with a baseline Eastern Cooperative Oncology Group Performance Status (ECOG PS) of 0 (*n* = 321; 48.8%) or 1 (*n* = 272; 41.3%). The majority of patients (*n* = 436; 66.3%) received ramucirumab as a second-line treatment, with 11.9% (*n* = 78) receiving ramucirumab as first-line treatment and 21.7% (*n* = 143) receiving ramucirumab as third-line or later treatment. Ramucirumab was received as late-line (≥ 3rd) therapy in 46.3% of patients in the monotherapy group and 15.5% patients in the ramucirumab + paclitaxel group. ECOG PS was ≥ 2 in 24.4% and 5.9% of patients in the monotherapy and ramucirumab + paclitaxel groups, respectively. Overall, 98.8% (*n* = 650) of patients had received prior systemic chemotherapy, including adjuvant therapy, for gastric cancer, (Online Resource 2), most commonly tegafur/gimeracil/oteracil potassium combination drug (*n* = 542; 83.4%), cisplatin (*n* = 282; 43.4%), and oxaliplatin (*n* = 268; 41.2%).

**Table 1:** Patient demographics and baseline clinical characteristics

Characteristic, *n* (%)	Ramucirumab monotherapy *N* = 123	Ramucirumab + paclitaxel *N* = 528	Overall analysis population *N* = 658^a^
Sex			
Male	84 (68.3)	375 (71.0)	463 (70.4)
Female	39 (31.7)	153 (29.0)	195 (29.6)
Age, years			
Median	69.0	68.0	68.0
Minimum–maximum	21–88	29–94	21–94
≥ 75 years	39 (31.7)	107 (20.3)	147 (22.3)
Body Mass Index, kg/m^2^			
Median	19.3	19.8	19.8
Minimum–maximum	12.3–28.3	12.9–32.9	12.3–32.9
HER2 status			
Positive	26 (21.1)	102 (19.3)	128 (19.5)
Negative	87 (70.7)	380 (72.0)	473 (71.9)
Unknown or not done	10 (8.1)	46 (8.7)	56 (8.5)
Not described	0 (0.0)	0 (0.0)	1 (0.2)
Primary tumor site			
Gastric	116 (94.3)	488 (92.4)	609 (92.6)
Gastroesophageal junction	7 (5.7)	40 (7.6)	48 (7.3)
Not described	0 (0.0)	0 (0.0)	1 (0.2)
Presence of residual primary tumor			
No	61 (49.6)	252 (47.7)	316 (48.0)
Yes	62 (50.4)	276 (52.3)	341 (51.8)
Not described	0 (0.0)	0 (0.0)	1 (0.2)
Metastasis and recurrent sites			
No	0 (0.0)	5 (1.0)	5 (0.8)
Yes	123 (100)	523 (99.1)	652 (99.1)
Peritoneal seeding (with ascites)	42 (34.2)	158 (30.2)	201 (30.8)
Peritoneal seeding (no ascites)	19 (15.5)	123 (23.5)	143 (21.9)
Lymph nodes (intraperitoneal)	62 (50.4)	273 (52.2)	337 (51.7)
Lymph nodes (other)	21 (17.1)	61 (11.7)	83 (12.7)
Liver	44 (35.8)	148 (28.3)	194 (29.8)
Lung	25 (20.3)	48 (9.2)	73 (11.2)
Bone	6 (4.9)	29 (5.5)	35 (5.4)
Brain	1 (0.8)	2 (0.4)	3 (0.5)
Other	13 (10.6)	63 (12.1)	76 (11.7)
Not described	0 (0.0)	0 (0.0)	1 (0.2)
Treatment line (ramucirumab)^b^			
1st	4 (3.3)	74 (14.0)	78 (11.9)
2nd	62 (50.4)	372 (70.5)	436 (66.3)
3rd	20 (16.3)	63 (11.9)	87 (13.2)
≥ 4th	37 (30.1)	19 (3.6)	56 (8.5)
Not described	0 (0.0)	0 (0.0)	1 (0.2)
Eastern cooperative oncology group Performance status			
0	38 (30.9)	281 (53.2)	321 (48.8)
1	53 (43.1)	215 (40.7)	272 (41.3)
2	26 (21.1)	28 (5.3)	54 (8.2)
≥ 3	4 (3.3)	3 (0.6)	7 (1.1)
Not done	2 (1.6)	1 (0.2)	4 (0.6)
Complications			
No	43 (35.0)	216 (40.9)	262 (39.8)
Yes	80 (65.0)	312 (59.1)	395 (60.0)
High blood pressure	33 (41.3)	145 (46.5)	181 (45.8)
Renal disease	10 (12.5)	60 (19.2)	70 (17.7)
Liver disease	6 (7.5)	26 (8.3)	32 (8.1)
Thromboembolism	3 (3.8)	22 (7.1)	25 (6.3)
Inflammatory digestive tract disease	0 (0.0)	3 (1.0)	3 (0.8)
Hemorrhagic diathesis and coagulation disorder	0 (0.0)	2 (0.6)	2 (0.5)
Wound healing disorder after major surgery	0 (0.0)	1 (0.3)	1 (0.3)
Other	61 (76.3)	208 (66.7)	270 (68.4)
Not described	0 (0.0)	0 (0.0)	1 (0.2)

*HER2* human epidermal growth factor receptor-2, *N* number of patients in population, *n* number of patients in category

^a^Includes two subgroups with data not shown separately due to small sample size: patients who received other ramucirumab combination therapies (*N* = 4) or unspecified ramucirumab therapy (*N* = 3)

^b^Treatment line as reported by the investigator

Mean (SD) duration of therapy was 19.9 (15.7) weeks (median: 16.0 weeks) for ramucirumab (Table 2). Overall, the mean (SD) dose intensity for ramucirumab was 3.4 (0.6) mg/kg/weeks (median 3.6 mg/kg/week) and mean (SD) relative dose intensity was 85.1% (16.0) (median: 89.3%). The mean duration of therapy (SD) was 11.1 (12.6) weeks in the monotherapy group and 21.9 (15.6) weeks in the ramucirumab + paclitaxel group. The mean (SD) relative dose intensity was 92.2% (13.1) in the monotherapy group and 83.5% (16.2) in the ramucirumab + paclitaxel group (Table 2).

**Table 2:** Dose duration and intensity of ramucirumab and paclitaxel

	Ramucirumab monotherapy *N* = 123	Ramucirumab + paclitaxel *N* = 528		Overall analysis population *N* = 658^a^
	Ramucirumab	Ramucirumab	Paclitaxel	Ramucirumab
Duration of therapy, weeks				
*n*	123	528	528	658
Mean (SD)	11.1 (12.6)	21.9 (15.6)	21.4 (15.3)	19.9 (15.7)
Median	6.3	18.0	17.9	16.0
Minimum–maximum	2.0–53.0	2.0–72.3	2.0–76.0	2.0–72.3
Dose intensity^b^, mg/kg/week				
*n*	123	527^c^	518^d^	656
Mean (SD)	3.7 (0.5)	3.3 (0.6)	40.1 (11.9)	3.4 (0.6)
Median	3.9	3.5	40.0	3.6
Minimum–maximum	1.8–4.4	1.1–4.7	6.8–96.7	1.1–4.7
Relative dose intensity^b^, %				
*n*	123	527^c^	518^d^	656
Mean (SD)	92.2 (13.1)	83.5 (16.2)	66.8 (19.8)	85.1 (16.0)
Median	97.8	86.9	66.7	89.3
Minimum–maximum	44.7–109.1	26.2–118.5	11.3–161.1	26.2–118.5

*N* number of patients in population, *n* number of patients in category, *SD* standard deviation

^a^Includes two subgroups with data not shown separately due to small sample size: patients who received other ramucirumab combination therapies (*N* = 4) or unspecified ramucirumab therapy (*N* = 3)

^b^Dose intensity refers to the actual amount of drug administered per week, calculated per patient as the total dose of ramucirumab (mg) per body weight (kg) per week of treatment. Relative dose intensity refers to the percentage of drug administered relative to the planned dose intensity

^c^One patient not included, as weight not provided

^d^Nine patients not included, as dose information provided was insufficient for dose intensity calculations

### Safety

**Fig. 1:**
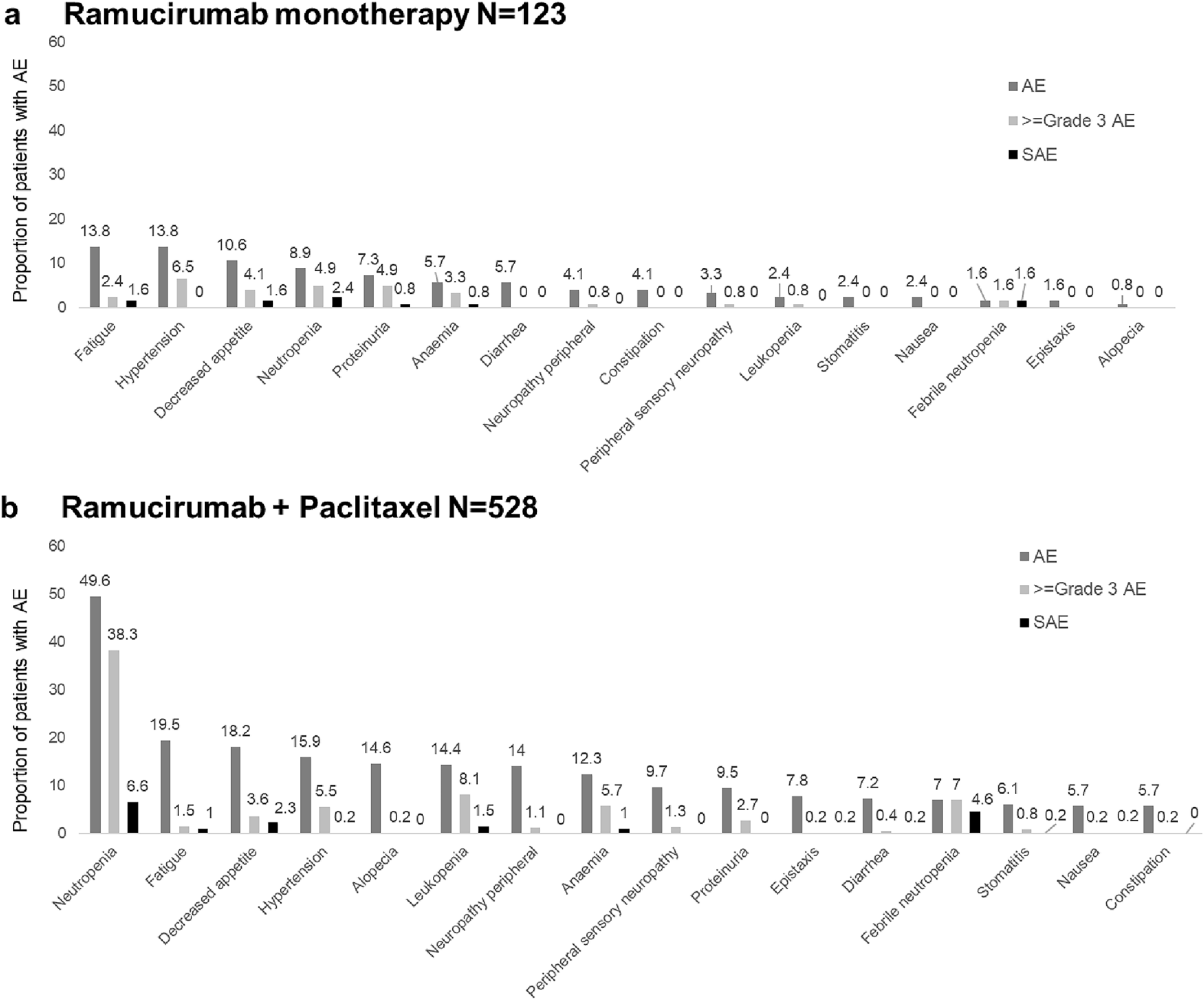
Adverse events by consolidated terms. AEs reported in ≥ 5% of patients in either the **a** ramucirumab monotherapy or **b** ramucirumab + paclitaxel combination therapy group are shown. Consolidated terms included the following Medical Dictionary for Regulatory Activities preferred terms (in parentheses) anemia (anemia, hemoglobin decreased); fatigue (fatigue, malaise, asthenia); leukopenia (leukopenia, white blood cell count decreased); neutropenia (neutropenia; neutrophil count decreased), and proteinuria (proteinuria, protein urine, protein urine present). *AE* adverse event, *SAE* serious adverse event

Overall, AEs were observed in 561 (85.3%) patients, and ≥ Grade 3 AEs were observed in 365 patients (55.5%). Neutropenia (*n* = 273; 41.5%), fatigue (*n* = 124, 18.8%), decreased appetite (*n* = 112; 17.0%), and hypertension (*n* = 103; 15.7%) were the most common AEs of any grade whereas neutropenia (*n* = 208; 31.6%), leukopenia (*n* = 44; 6.7%), and febrile neutropenia (*n* = 39; 5.9%) were the most common ≥ Grade 3 AEs. The majority of patients reported ≥ 1 AE (monotherapy: any grade, 77/123; 62.6%; ≥ Grade 3, 42/123; 34.2%; ramucirumab + paclitaxel: any grade, 479/528; 90.7%; ≥ Grade 3, 321/528; 60.8%; Fig. 1). AEs commonly known to occur with paclitaxel were reported in the ramucirumab + paclitaxel group (e.g., neutropenia: any grade, 49.6%; ≥ Grade 3, 38.3%; leukopenia: any grade, 14.4%; ≥ Grade 3, 8.1%; peripheral neuropathy: any grade, 14.0%; ≥ Grade 3, 1.1%; alopecia: any grade, 14.6%; ≥ Grade, 3 0.2%) and were numerically less frequent in the monotherapy group (neutropenia: any grade, 8.9%; ≥ Grade 3, 4.9%; leukopenia: any grade, 2.5%; ≥ Grade 3, 0.8%; peripheral neuropathy: any grade, 4.1%; ≥ Grade 3, 0.8%; alopecia: any grade, 0.8%; ≥ Grade 3, 0%; Fig. 1). The majority of common AEs (≥ 70%) were reported as resolved or resolving by data cutoff at the end of the study period (Online Resource 3). Overall, the incidence rates of interstitial lung disease (ILD) and pneumonitis were 0.9% (monotherapy, *n* = 1; ramucirumab + paclitaxel, *n* = 5) and 0.8% (ramucirumab + paclitaxel, *n* = 5), respectively, all of which were ≤ Grade 3.

SAEs were reported in 153 (23.3%) patients (monotherapy: 23/123; 18.7%; ramucirumab + paclitaxel: 129/528; 24.4%). The most commonly reported SAEs in both the ramucirumab monotherapy and ramucirumab + paclitaxel groups were neutropenia (monotherapy: *n* = 3; 2.4%; ramucirumab + paclitaxel: *n* = 35; 6.6%), febrile neutropenia (monotherapy: *n* = 2; 1.6%; ramucirumab + paclitaxel: *n* =  24; 4.6%), and decreased appetite (monotherapy: *n* = 2; 1.6%; ramucirumab + paclitaxel: *n* = 12; 2.3%; Fig. 1). The incidence of SAEs of ILD and pneumonitis was 0.6% (*n* = 3) and 0.2% (*n* = 1), respectively, in the ramucirumab + paclitaxel group and 0.8% (*n* = 1) for ILD in the monotherapy group. Grade 5 AEs were reported for 4 patients (*n* = 1 each), including metastases to meninges and gastric perforation in the monotherapy group and pneumonia aspiration and death in the ramucirumab + paclitaxel group. Gastric perforation was the only grade 5 AE deemed related to ramucirumab. The patient experienced gastric perforation at the location of the primary tumor 2 weeks after starting ramucirumab, and the cause of death was reported as disease progression with bleeding from the perforation site.

AESIs for ramucirumab are summarized by treatment in Online Resource 4. Overall, patients most commonly experienced AESIs of hypertension (any grade *n* = 103; 15.7%; ≥ Grade 3 *n* = 37, 5.6%), bleeding/hemorrhage events (any grade *n* = 73, 11.1%; ≥ Grade 3 *n* = 13, 2%), including gastrointestinal hemorrhage events (any grade *n* = 17, 2.6%; ≥ Grade 3 *n* = 9, 1.4%), proteinuria (any grade *n* = 60; 9.1%; ≥ Grade 3 *n* = 20, 3.0%), and liver injury/liver failure (any grade *n* = 39; 5.9%; ≥ Grade 3 *n* = 9, 1.4%).

**Fig. 2:**
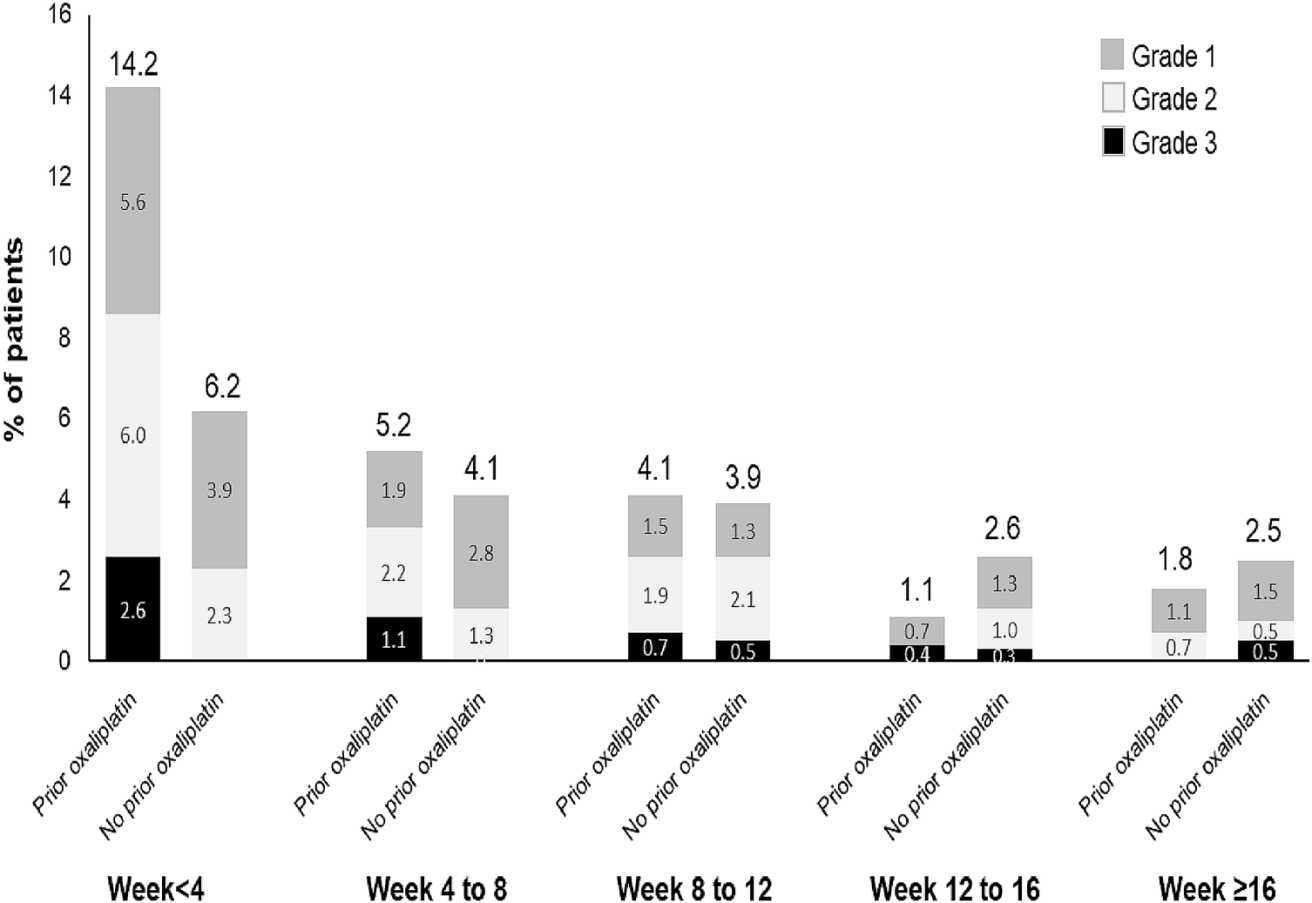
Incidence of nervous system disorders across cycles stratified by prior oxaliplatin. The proportion of patients reporting nervous system disorders, shown across ramucirumab treatment cycles (weeks) and by the worst grade of adverse event that occurred in each patient

The incidence of AEs by baseline clinical characteristics is shown in Online Resource 5. The incidence of AEs was not found to differ substantially by primary tumor site, metastatic or recurrent tumor site, or advanced age (≥ 75). As oxaliplatin is associated with acute and cumulative neurotoxicity [15], we also compared AE incidence of patients with and without prior oxaliplatin treatment. Over the study period, total AE incidence was similar between the two groups (prior oxaliplatin, 231/268, 86.1%; no prior oxaliplatin, 329/389; 84.6%), but the incidence of nervous system disorders was higher in the prior oxaliplatin group (69/268; 25.7%) compared with the group without prior oxaliplatin (73/389; 18.8%). The difference in the incidence of nervous system disorders was evident in all grades (grade 1–3) of AEs during the first 4 weeks of ramucirumab treatment but not at later treatment cycles (Fig. 2).

### Treatment discontinuation

Overall, 490 of 658 patients (74.5%) had discontinued ramucirumab treatment by 6 months, with 168 patients (25.5%) continuing treatment (Table 3). At 12 months, an additional 119 patients had discontinued (accumulated discontinuation 0–12 months: 92.6%), with 48 (7.3%) still on treatment at the end of the study. A numerically greater proportion of patients in the ramucirumab monotherapy group discontinued compared to the ramucirumab + paclitaxel group (accumulated discontinuation 0–12 months: 98.4% versus 91.5%, respectively; Table 3). The reasons for discontinuation were similar across treatment groups, most commonly progressive disease (75.9%) and AEs (13.0%; Table 3). The most common AEs leading to discontinuation were anemia (*n* = 12; 15.2%), neutrophil count decreased (*n* = 10; 12.7%), febrile neutropenia (*n* = 6; 7.6%), hypertension (*n* = 6; 7.6%), and alopecia (*n* = 5; 6.3%). Five patients discontinued due to neuropathy, all of whom had received prior oxaliplatin (5/268; 1.9%).

**Table 3:** Discontinuation of ramucirumab

	Ramucirumab monotherapy *N* = 123	Ramucirumab + paclitaxel *N* = 528	Overall analysis population *N* = 658^a^
Status at 6 months *n* (%)			
Continuing	10 (8.1)	154 (29.2)	168 (25.5)
Discontinued	113 (91.9)	374 (70.8)	490 (74.5)
Status at 12 months *n* (%)			
Continuing	2 (1.6)	45 (8.5)	48 (7.3)
Discontinued	8 (6.5)	109 (20.6)	119 (18.1)
Not described	0 (0.0)	0 (0.0)	1 (0.2)
Reason for discontinuation			
Progressive disease	89 (73.6)	369 (76.4)	462 (75.9)
Adverse event	16 (13.2)	63 (13.0)	79 (13.0)
Physician decision	7 (5.8)	20 (4.1)	28 (4.6)
Patient decision	5 (4.1)	19 (3.9)	24 (3.9)
Death	2 (1.7)	8 (1.7)	10 (1.6)
Lost to follow-up	1 (0.8)	2 (0.4)	3 (0.5)
Symptom improvement	0 (0.0)	2 (0.4)	2 (0.3)
No visits after enrollment	1 (0.8)	0 (0.0)	1 (0.2)

*N* number of patients in population, *n* number of patients in category

^a^Includes two subgroups with data not shown separately due to small sample size: patients who received other ramucirumab combination therapies (*N* = 4) or unspecified ramucirumab therapy (*N* = 3)

### Effectiveness

**Fig. 3:**
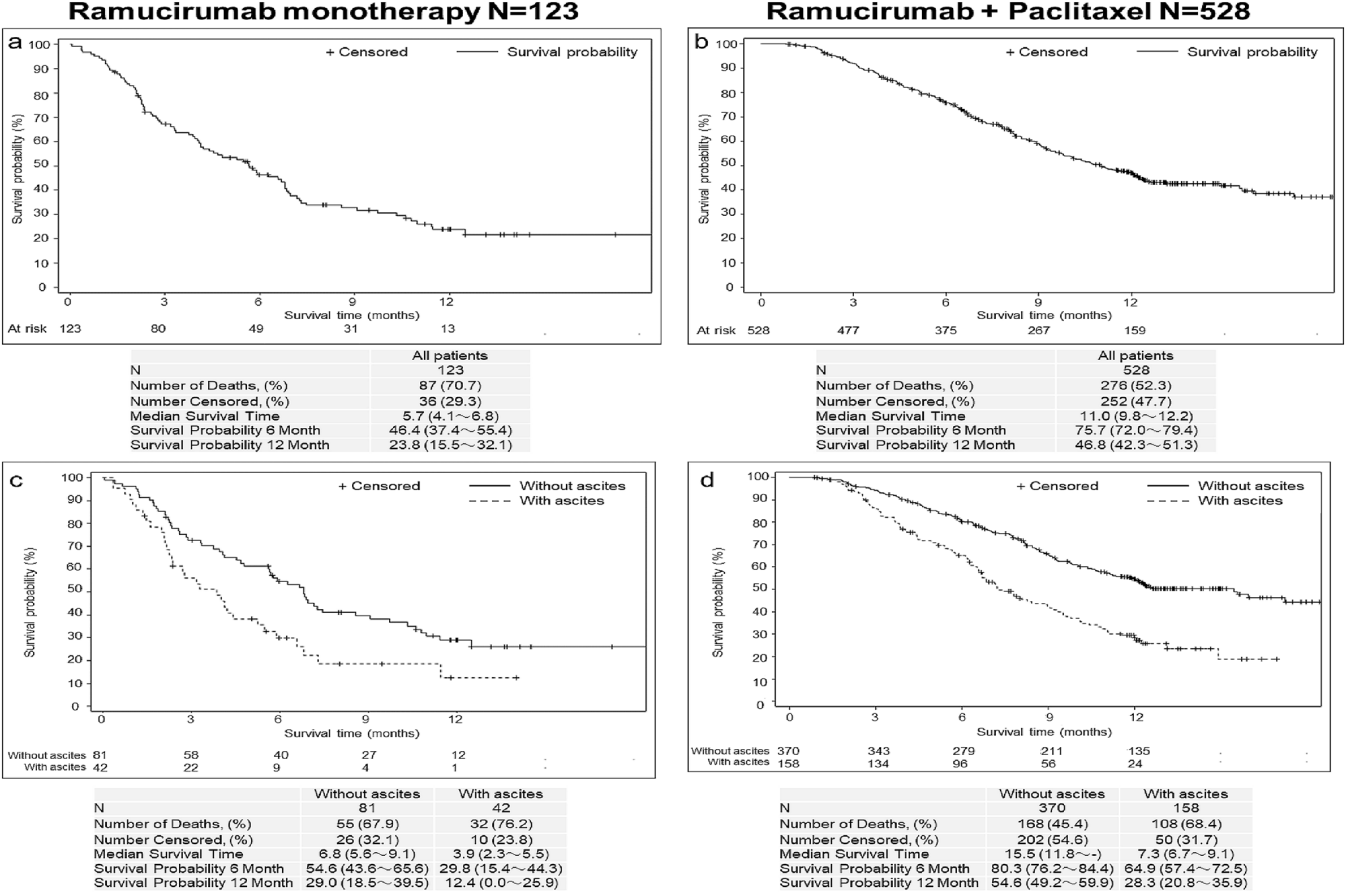
Survival curves. Kaplan–Meier survival curves shown by treatment category (**a**, **b**) and by the presence and absence of ascites (**c**, **d**). Median survival time (months) and 6- and 12-month survival rates (as percentage) are shown with 95% confidence intervals. - not reached, *N* number of patients in population

At the time of database lock, 55.3% of patients had died in the overall analysis population (364/658 patients), and death was most frequently due to the primary disease (gastric cancer; 98.6%; 359/364 patients). Median survival time was 5.7 months (95% CI 4.1–6.8 months) in the monotherapy group and 11.0 months (95% CI 9.8–12.2 months; Fig. 3a and b) in the ramucirumab + paclitaxel group. Patients with ascites had lower median survival (monotherapy, with ascites: 3.9 months; without ascites: 6.8 months; ramucirumab + paclitaxel, with ascites: 7.3 months; without ascites: 15.5 months; Fig. 3c and d). Median survival was similar between patients aged < 75 years and ≥ 75 years (monotherapy: < 75 years: 5.3 months; ≥ 75 years: 6.3 months; ramucirumab + paclitaxel: < 75 years: 11.2 months; ≥ 75 years: 10.4 months; Online Resource 6).

## Discussion

The current analysis was conducted to provide real-world data on the safety and effectiveness of ramucirumab in patients with advanced gastric cancer in Japan. Our results confirm that the safety profile for ramucirumab in patients under routine clinical care in Japan is in accordance with that observed in prior clinical trials, with no new safety concerns identified. Overall, AEs were reported in 85.3% of patients, with 55.5% experiencing ≥ Grade 3 AEs and 23.3% experiencing SAEs. AEs of blood and lymphatic disorders, nervous system disorders, and gastrointestinal disorders were the most frequently reported. The most common SAEs included neutropenia, febrile neutropenia, and decreased appetite. Hypertension was the most common AESI, reported by 15.7% of patients, although an SAE of hypertension occurred in only 1 patient. AEs were generally manageable, with most patients discontinuing treatment due to progressive disease (75.9%) rather than an AE (13.0%). AEs in a majority of patients were reported as resolved or resolving at the end of the study period.

Ramucirumab monotherapy was associated with numerically worse baseline ECOG PS and later line of ramucirumab treatment compared with ramucirumab + paclitaxel therapy. The monotherapy group also had a numerically shorter duration of treatment, higher rate of discontinuation, and shorter survival compared with the ramucirumab + paclitaxel therapy group. These differences suggest that patients who received ramucirumab monotherapy may have been in poorer health, and therefore, were not candidates for ramucirumab + paclitaxel combination therapy. AEs were consistent with the known AE profiles for each drug, with patients in the ramucirumab + paclitaxel group experiencing a numerically greater number of AEs known to be adverse drug reactions for paclitaxel.

Nearly all patients (98.8%) had received prior systemic chemotherapy for gastric cancer, which most commonly included tegafur/gimeracil/oteracil potassium and platinum-based drug combinations. Platinum-based drug usage is of particular interest in that peripheral neuropathy and other neurotoxicities are well established, treatment-related AEs associated with oxaliplatin and other platinum-based therapies [16, 17]. In the current study, the overall incidence of AEs of the nervous system was slightly higher in patients with prior oxaliplatin (25.7%) compared with those without prior oxaliplatin (18.8%), with discontinuations due to neuropathy only occurring in the prior oxaliplatin group. Differences between the two groups in the incidence of AEs of neuropathy were most evident during the first 4 weeks of ramucirumab treatment, suggesting that prior oxaliplatin had a limited effect on subsequent ramucirumab treatment in terms of neuropathies.

In the current study, median survival time was 11.0 months for the ramucirumab + paclitaxel group and 5.7 months for the ramucirumab monotherapy group, which demonstrates effectiveness for ramucirumab in line with two prior clinical study studies in global [5, 6] and Japanese populations [18, 19]. Advanced age (≥ 75 years) affected neither survival nor AE incidence rates, indicating that ramucirumab retained effectiveness in elderly patients with gastric cancer without increasing the risk of AEs. In contrast, the presence of ascites was associated with shorter median survival times in both the ramucirumab monotherapy and ramucirumab + paclitaxel groups, which is consistent with prior studies showing poorer prognosis for gastric cancer with malignant ascites [20]. In the phase 3 RAINBOW study, a multivariate analysis using a stepwise Cox model also identified the presence of ascites as a strong independent predictor of survival, with median overall survival times in the placebo group lower in patients with ascites (5.2 months) than in those without ascites (8.5 months) [6, 21]. Nevertheless, ramucirumab + paclitaxel improved survival in patients with gastric cancer both with and without ascites, extending median overall survival by 2.0 and 2.9 months compared with placebo, respectively [21].

The strengths of this study include its prospective design and inclusion of a heterogeneous Japanese population. In addition, the majority of patients in this study received prior tegafur/gimeracil/oteracil potassium, which is commonly used in Japan but not elsewhere, and this increases the value of the current analysis for Japan. However, important limitations inherent to post-marketing surveillance studies should be considered which limit the conclusions that can be drawn, including the lack of a control group, non-interventional design, and low numbers included for AE subgroup analyses. In addition, although separate analyses for ramucirumab monotherapy and combination therapy provided new information on the effectiveness and safety of these regimens in Japanese patients, these results should be interpreted carefully due to the lack of statistical testing of differences and covariate adjustment between subgroups. Furthermore, as the current study had no specific exclusions in the patient population or restrictions on treatment history/concomitant medications, the current data are not directly comparable to the data from prior clinical trials, which had strict sets of eligibility criteria. It is also recognized that AE incidence is generally lower in post-marketing studies compared with clinical trials, as physicians do not have the same obligations to report AEs in post-marketing studies, which lack the structured data-capturing systems of clinical trials. Nevertheless, with these limitations in mind, it is worth noting that the survival benefit and safety profile in the current real-world setting were broadly similar to the outcomes of prior clinical trials [5, 6, 18, 19]. In Japanese patients who received ramucirumab in the phase 3 RAINBOW study, the incidence of AEs was 100% and 83.8% for any grade and ≥ Grade 3 AEs, respectively, with neutropenia and lymphopenia the most common AEs reported, and median overall survival was 11.4 months [18].

## Conclusion

This study adds to the limited existing data on ramucirumab in patients with unresectable advanced or recurrent gastric cancer under routine clinical practice, demonstrating that effectiveness and safety outcomes in the real-world setting are consistent with the findings of clinical studies.

## Supplementary Information

Below is the link to the electronic supplementary material.

Supplementary file1 (PDF 365 kb)
