# Supplementary material for: A post-marketing observational study of ramucirumab in patients with gastric cancer in Japan
Source: Gastric Cancer. 2021 May 28;24(6):1320–9. doi: 10.1007/s10120-021-01199-0 (PMC8502135; doi:10.1007/s10120-021-01199-0)
Supplement: Supplementary file 1 — Supplementary file1 (PDF 365 kb) [file 10120_2021_1199_MOESM1_ESM.pdf]

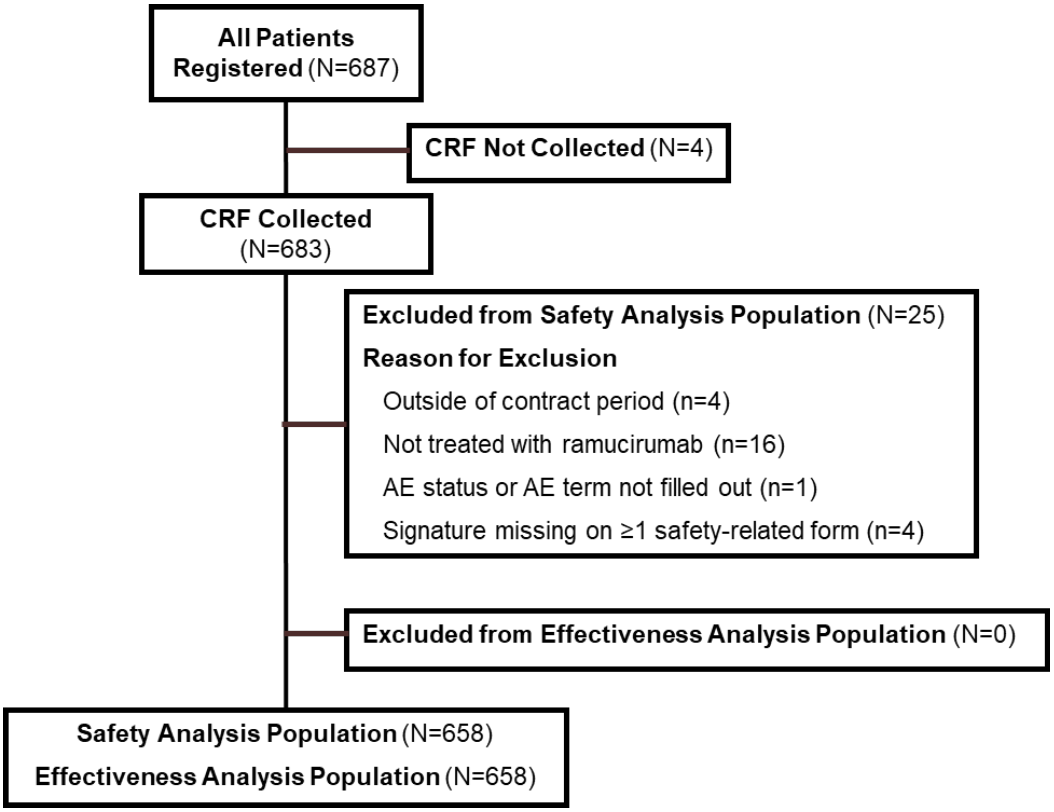

Online Resource 1. Patient disposition. AE, adverse event; CRF, case report form; N, number of patients; n, number of patients in category.

Online Resource 2. Prior treatment of gastric cancer with chemotherapy (including adjuvant therapy)

Prior anti-cancer drug[a]		Ramucirumab Monotherapy N=123	Ramucirumab + Paclitaxel N=528	Overall Analysis Population N=658[b]
**Prior therapy n (%)**	No	0 (0.0)	7 (1.3)	7 (1.1)
	Yes	123 (100)	521 (98.7)	650 (98.8)
	Not described	0 (0.0)	0 (0.0)	1 (0.2)
**Drug name (≥10 patients)**	Tegafur/Gimeracil/Oteracil potassium combined drug	104	433	542
	Cisplatin	53	226	282
	Oxaliplatin	52	213	268
	Paclitaxel	68	96	167
	Capecitabine	29	122	152
	Trastuzumab (Genetic recombination)	19	86	105
	Irinotecan hydrochloride hydrate	44	37	82
	Docetaxel	16	49	65
	Paclitaxel (albumin-bound)	19	10	30
	Antineoplastic agents, N.E.C.	7	11	19
	Fluorouracil	5	12	17

[a]Patients were counted in all applicable categories.
[b]Includes two subgroups with data not shown separately due to small sample size: patients who received other ramucirumab combination therapies (N=4) or unspecified ramucirumab therapy (N=3).

N, number of patients in safety population; n, number of patients in category.

Online Resource 3. Outcomes for adverse events by MedDRA System Organ Class and Preferred Terms in overall analysis population

		Outcomes					
**Overall Analysis Population, N=658, n (%)**	**Total events**	**Recovered**	**Recovering**	**Not recovered**	**Recovered with sequelae**	**Fatal**	**Unknown**
**System Organ Class**[a]							
Preferred Terms, reported in ≥5.0% patients							
**Blood and lymphatic system disorders**	170	83 (48.8)	68 (40.0)	18 (10.6)	1 (0.6)	0 (0.0)	0 (0.0)
Anaemia	67	12 (17.9)	37 (55.2)	17 (25.4)	1 (1.5)	0 (0.0)	0 (0.0)
Febrile neutropenia	41	33 (80.5)	8 (19.5)	0 (0.0)	0 (0.0)	0 (0.0)	0 (0.0)
Leukopenia	10	5 (50.0)	5 (50.0)	0 (0.0)	0 (0.0)	0 (0.0)	0 (0.0)
Neutropenia	50	33 (66.0)	17 (34.0)	0 (0.0)	0 (0.0)	0 (0.0)	0 (0.0)
**Cardiac disorders**	6	2 (33.3)	1 (16.7)	3 (50.0)	0 (0.0)	0 (0.0)	0 (0.0)
**Congenital disorders**	1	0 (0.0)	1 (100)	0 (0.0)	0 (0.0)	0 (0.0)	0 (0.0)
**Ear and labyrinth disorders**	2	1 (50.0)	0 (0.0)	1 (50.0)	0 (0.0)	0 (0.0)	0 (0.0)
**Eye disorders**	2	1 (50.0)	1 (50.0)	0 (0.0)	0 (0.0)	0 (0.0)	0 (0.0)
**Gastrointestinal disorders**	282	119 (42.2)	96 (34.0)	53 (18.8)	3 (1.1)	1 (0.4)	10 (13.6)
Constipation	41	13 (31.7)	19 (46.3)	8 (19.5)	0 (0.0)	0 (0.0)	1 (2.4)
Diarrhea	57	31 (54.4)	16 (28.1)	8 (14.0)	0 (0.0)	0 (0.0)	2 (3.5)
Nausea	38	23 (60.5)	5 (13.2)	10 (26.3)	0 (0.0)	0 (0.0)	0 (0.0)
Stomatitis	38	13 (34.2)	13 (34.2)	8 (21.1)	0 (0.0)	0 (0.0)	4 (10.5)
**General disorders and administration site conditions**	225	63 (28.0)	73 (32.4)	83 (36.9)	0 (0.0)	1 (0.4)	5 (2.2)
Fatigue	68	14 (20.6)	18 (26.5)	32 (47.1)	0 (0.0)	0 (0.0)	4 (5.9)
Malaise	67	13 (19.4)	32 (47.8)	22 (32.8)	0 (0.0)	0 (0.0)	0 (0.0)
**Hepatobiliary disorders**	20	5 (25.0)	8 (40.0)	7 (35.0)	0 (0.0)	0 (0.0)	0 (0.0)
**Immune system disorders**	5	3 (60.0)	2 (40.0)	0 (0.0)	0 (0.0)	0 (0.0)	0 (0.0)
**Infections and infestations**	60	32 (53.3)	25 (41.7)	3 (5.0)	0 (0.0)	0 (0.0)	0 (0.0)

**Injury, poisoning, and procedural complications**	11	6 (54.6)	4 (36.4)	0 (0.0)	0 (0.0)	0 (0.0)	1 (9.1)
**Investigations**	653	377 (57.7)	215 (32.9)	58 (8.9)	0 (0.0)	0 (0.0)	3 (0.5)
Haemoglobin decreased	10	1 (10.0)	5 (50.0)	4 (40.0)	0 (0.0)	0 (0.0)	0 (0.0)
Neutrophil count decreased	435	273 (62.8)	141 (32.4)	19 (4.4)	0 (0.0)	0 (0.0)	2 (0.5)
Protein urine	18	4 (22.2)	9 (50.0)	5 (27.8)	0 (0.0)	0 (0.0)	0 (0.0)
Protein urine present	1	0 (0.0)	1 (100)	0 (0.0)	0 (0.0)	0 (0.0)	0 (0.0)
White blood cell count decreased	90	64 (71.1)	20 (22.2)	5 (5.6)	0 (0.0)	0 (0.0)	1 (1.1)
**Metabolism and nutrition disorders**	156	52 (33.3)	57 (36.5)	45 (28.9)	0 (0.0)	0 (0.0)	2 (1.3)
Decreased appetite	126	35 (27.8)	50 (39.7)	39 (31.0)	0 (0.0)	0 (0.0)	2 (1.6)
**Musculoskeletal and connective tissue disorders**	21	7 (33.3)	7 (33.3)	7 (33.3)	0 (0.0)	0 (0.0)	0 (0.0)
**Neoplasms-benign, malignant, or unspecified**	8	1 (12.5)	4 (50.0)	2 (25.0)	0 (0.0)	1 (12.5)	0 (0.0)
**Nervous system disorders**	183	18 (9.8)	52 (28.4)	103 (56.3)	3 (1.6)	0 (0.0)	7 (3.8)
Neuropathy peripheral	82	4 (4.9)	22 (26.8)	53 (64.6)	1 (1.2)	0 (0.0)	2 (2.4)
Peripheral sensory neuropathy	59	5 (8.5)	13 (22.0)	35 (59.3)	2 (3.4)	0 (0.0)	4 (6.8)
**Psychiatric disorders**	2	0 (0.0)	2 (100)	0 (0.0)	0 (0.0)	0 (0.0)	0 (0.0)
**Renal and urinary disorders**	61	17 (27.9)	27 (44.3)	12 (19.7)	0 (0.0)	0 (0.0)	5 (8.2)
Proteinuria	51	15 (29.4)	21 (41.2)	10 (19.6)	0 (0.0)	0 (0.0)	5 (9.8)
**Respiratory, thoracic, and mediastinal disorders**	83	31 (37.4)	32 (38.6)	17 (20.5)	2 (2.4)	1 (1.2)	0 (0.0)
**Skin and subcutaneous tissue disorders**	116	17 (14.7)	33 (28.5)	59 (50.9)	0 (0.0)	0 (0.0)	7 (6.0)
**Vascular disorders**	112	26 (23.2)	53 (47.3)	31 (27.7)	0 (0.0)	0 (0.0)	2 (1.8)
Hypertension	107	25 (23.4)	51 (47.7)	29 (27.1)	0 (0.0)	0 (0.0)	2 (1.9)

[a]MedDRA, version 21.1.

MedDRA, Medical Dictionary for Regulatory Activities; N, number of patients in safety population; n, number of events

Online Resource 4. Summary of ramucirumab AESIs by grade and seriousness

	Ramucirumab monotherapy N=123			Ramucirumab + Paclitaxel N=528			Overall Analysis Population N=658[a]		
		≥Grade 3			≥Grade 3			≥Grade 3	
	Any AE n (%)	AE n (%)	SAE n (%)	Any AE n (%)	AE n (%)	SAE n (%)	Any AE n (%)	AE n (%)	SAE n (%)
**Liver Injury / liver failure**	6 (4.9)	2 (1.6)	1 (0.8)	33 (6.3)	7 (1.3)	2 (0.4)	39 (5.9)	9 (1.4)	3 (0.5)
Aspartate aminotransferase increased	5 (4.1)	1 (0.8)	0 (0.0)	19 (3.6)	1 (0.2)	0 (0.0)	24 (3.7)	2 (0.3)	0 (0.0)
Alanine aminotransferase increased	4 (3.3)	0 (0.0)	0 (0.0)	15 (2.8)	0 (0.0)	0 (0.0)	19 (2.9)	0 (0.0)	0 (0.0)
Hepatic function abnormal	0 (0.0)	0 (0.0)	0 (0.0)	8 (1.5)	5 (1.0)	2 (0.4)	8 (1.2)	5 (0.8)	2 (0.3)
Hyperbilirubinaemia	0 (0.0)	0 (0.0)	0 (0.0)	2 (0.4)	0 (0.0)	0 (0.0)	2 (0.3)	0 (0.0)	0 (0.0)
Drug-induced liver injury	0 (0.0)	0 (0.0)	0 (0.0)	1 (0.2)	1 (0.2)	0 (0.0)	1 (0.2)	1 (0.2)	0 (0.0)
Gamma-glutamyltransferase increased	0 (0.0)	0 (0.0)	0 (0.0)	1 (0.2)	0 (0.0)	0 (0.0)	1 (0.2)	0 (0.0)	0 (0.0)
Jaundice	1 (0.8)	1 (0.8)	1 (0.8)	0 (0.0)	0 (0.0)	0 (0.0)	1 (0.2)	1 (0.2)	0 (0.0))
**Bleeding / hemorrhage events**[c]	7 (5.7)	3 (2.4)	3 (2.4)	66 (12.5)	10 (1.9)	9 (1.7)	73 (11.1)	13 (2.0)	12 (1.8)
**Gastrointestinal hemorrhage events**	3 (2.4)	2 (1.6)	2 (1.6)	14 (2.7)	4 (0.8	5 (1.0)	17 (2.6)	9 (1.4)	7 (1.1)
Gastric haemorrhage	1 (0.8)	1 (0.8)	1 (0.8)	5 (1.0)	1 (0.2)	3 (0.6)	6 (0.9)	5 (0.8)	4 (0.6)
Gastrointestinal haemorrhage	1 (0.8)	0 (0.0)	0 (0.0)	2 (0.4)	1 (0.2)	1 (0.2)	3 (0.5)	1 (0.2)	1 (0.2)
Upper gastrointestinal haemorrhage	1 (0.8)	1 (0.8)	1 (0.8)	2 (0.4)	1 (0.2)	1 (0.2)	3 (0.5)	2 (0.3)	2 (0.3)

Anal haemorrhage	0 (0.0)	0 (0.0)	0 (0.0)	1 (0.2)	0 (0.0)	0 (0.0)	1 (0.2)	0 (0.0)	0 (0.0)
Anastomotic haemorrhage	0 (0.0)	0 (0.0)	0 (0.0)	1 (0.2)	0 (0.0)	0 (0.0)	1 (0.2)	0 (0.0)	0 (0.0)
Haemorrhoidal haemorrhage	0 (0.0)	0 (0.0)	0 (0.0)	1 (0.2)	0 (0.0)	0 (0.0)	1 (0.2)	0 (0.0)	0 (0.0)
Lower gastrointestinal haemorrhage	0 (0.0)	0 (0.0)	0 (0.0)	1 (0.2)	1 (0.2)	0 (0.0)	1 (0.2)	1 (0.2)	0 (0.0)
Melaena	0 (0.0)	0 (0.0)	0 (0.0)	1 (0.2)	0 (0.0)	0 (0.0)	1 (0.2)	0 (0.0)	0 (0.0)
**Gastrointestinal perforation**	1 (0.8)	1 (0.8)	1 (0.8)	4 (0.8)	4 (0.8)	4 (0.8)	5 (0.8)	5 (0.8)	5 (0.8)
Gastric perforation	1 (0.8)	1 (0.8)	1 (0.8)	1 (0.2)	1 (0.2)	1 (0.2)	2 (0.3)	2 (0.3)	2 (0.3)
Gastrointestinal perforation	0 (0.0)	0 (0.0)	0 (0.0)	3 (0.6)	3 (0.6)	3 (0.6)	3 (0.5)	3 (0.5)	3 (0.5)
**Hypertension**	17 (13.8)	8 (6.5)	0 (0.0)	84 (15.9)	29 (5.5)	1 (0.2)	103 (15.7)	37 (5.6)	1 (0.2)
**Proteinuria**	9 (7.3)	6 (4.9)	1 (0.8)	50 (9.5)	14 (2.7)	0 (0.0)	60 (9.1)	20 (3.0)	1 (0.2)
**Arterial thromboembolic events**	0 (0.0)	0 (0.0)	0 (0.0)	4 (0.8)	2 (0.4)	4 (0.8)	4 (0.6)	2 (0.3)	4 (0.6)
**Venous thromboembolic events**	0 (0.0)	0 (0.0)	0 (0.0)	3 (0.6)	2 (0.4)	1 (0.2)	3 (0.5)	2 (0.3)	1 (0.2)
**Infusion related reaction**	5 (4.1)	2 (1.6)	2 (1.6)	2 (0.4)	0 (0.0)	0 (0.0)	7 (1.1)	2 (0.3)	2 (0.3)
**Congestive heart failure**	1 (0.8)	1 (0.8)	1 (0.8))	1 (0.2)	1 (0.2)	0 (0.0)	2 (0.3)	2 (0.3)	1 (0.2)
**Wound healing complications**	0 (0.0)	0 (0.0)	0 (0.0)	0 (0.0)	0 (0.0)	0 (0.0)	0 (0.0)	0 (0.0)	0 (0.0)
**Fistula**	0 (0.0)	0 (0.0)	0 (0.0)	1 (0.2)	0 (0.0)	1 (0.2)	1 (0.2)	0 (0.0)	1 (0.2)
**Reversible posterior leukoencephalopathy syndrome**	0 (0.0)	0 (0.0)	0 (0.0)	0 (0.0)	0 (0.0)	0 (0.0)	0 (0.0)	0 (0.0)	0 (0.0)

[a]Includes two subgroups with data not shown separately due to small sample size: patients who received other ramucirumab combination therapies (N=4) or unspecified ramucirumab therapy (N=3).

[b]Medical Dictionary for Regulatory Activities, version 21.1.
[c]Category includes gastrointestinal hemorrhage events.

AE, adverse event; AESI, adverse event of special interest; N, number of patients in analysis population; n, number of patients in category; SAE, serious adverse event.

Online Resource 5. Adverse events by patient characteristic

Characteristic	Subcategory	Adverse Events (Safety population N=658)		
		Nx	n	%
**Age (years)**	<75	511	432	84.5
	≥75	147	129	87.8
**Primary site**	Gastric	609	518	85.1
	Gastroesophageal junction	48	42	87.5
	Not described	1	1	100
**Metastasis and recurrent sites**	No	5	4	80.0
	Yes	652	556	85.3

N, number of patients in safety population; n, number of patients with adverse events; Nx, number of patients with non-missing information.

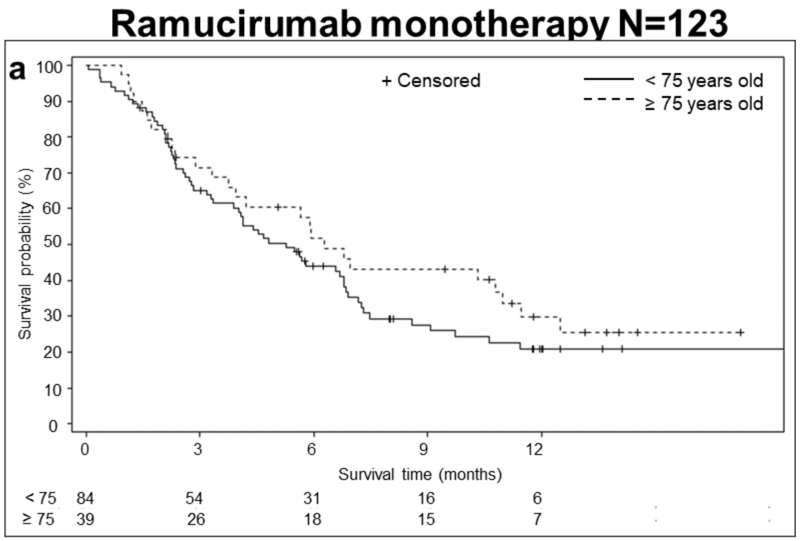

	< 75 years old	≥ 75 years old
N	84	39
Number of Deaths, (%)	61 (72.6)	26 (66.7)
Number Censored, (%)	23 (27.4)	13 (33.3)
Median Survival Time	5.3 (3.9～6.8)	6.3 (3.9～11.0)
Survival Probability 6 Month	44.0 (33.2～54.8)	51.8 (35.6～68.0)
Survival Probability 12 Month	20.7 (11.0～30.4)	29.7 (14.0～45.4)

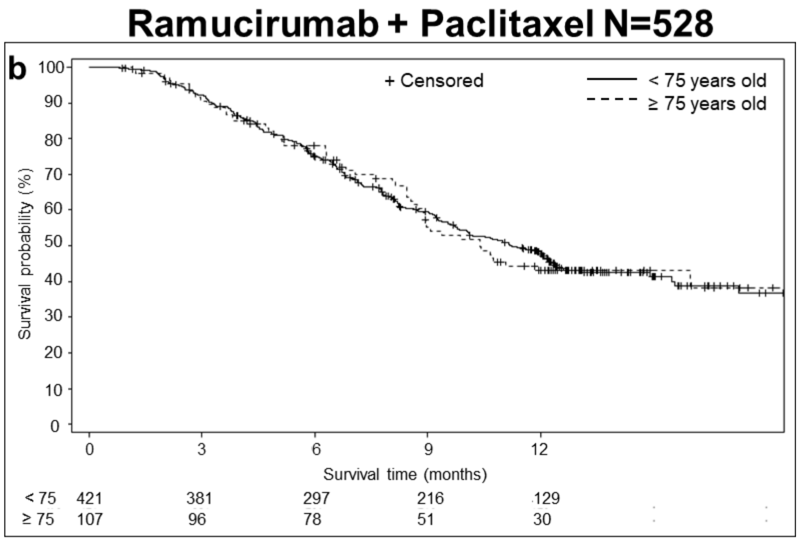

	< 75 years old	≥ 75 years old
N	421	107
Number of Deaths, (%)	219 (52.0)	57 (53.3)
Number Censored, (%)	202 (48.0)	50 (46.7)
Median Survival Time	11.2 (9.8～12.4)	10.4 (8.9～-)
Survival Probability 6 Month	75.1 (70.9～79.3)	78.1 (70.2～86.0)
Survival Probability 12 Month	47.8 (42.8～52.9)	43.0 (33.1～53.0)

Online Resource 6. Survival curve by age category. Survival curves generated using the Kaplan-Meier method. Median survival time (months) and 6- and 12-month survival rates (as percentage) are shown with 95% confidence intervals for patients aged <75 years old and ≥75 years old who have been treated with a) ramucirumab monotherapy; b) ramucirumab+paclitaxel combination therapy. -, not reached; N, number of patients in analysis population.
